# MRI Discriminates Thrombus Composition and ST Resolution after Percutaneous Coronary Intervention in Patients with ST-Elevation Myocardial Infarction

**DOI:** 10.1371/journal.pone.0018459

**Published:** 2011-04-08

**Authors:** Ignasi Barba, Bruno Garcia del Blanco, Omar Abdul-Jawad, José A. Barrabés, Gerard Martí, Enric Domingo, Joan Angel, David Garcia-Dorado

**Affiliations:** Department of Cardiology, Vall d'Hebron University Hospital and Research Institute, Universitat Autònoma de Barcelona, Barcelona, Spain; University of Modena and Reggio Emilia, Italy

## Abstract

Histological composition of material obtained by thrombus aspiration during percutaneous coronary intervention (PCI) in patients with ST-segment elevation acute myocardial infarction (STEMI) is highly variable. We aimed to characterize this material using magnetic resonance imaging (MRI) and to correlate MRI findings with the success of PCI in terms of ST-segment resolution. Thrombus aspiration during primary or rescue PCI was attempted in 100 consecutive STEMI patients, of whom enough material for MRI was obtained in 59. MR images were obtained at 9.4T and T1 and T2 values were measured. Patients with (n = 31) and without (n = 28) adequate ST resolution 120 min after PCI (≥70% of pre-PCI value) had similar baseline characteristics except for a higher prevalence of diabetes mellitus in the latter (10 vs. 43%, p = 0.003). T1 values were similar in both groups (1248±112 vs. 1307±85 ms, respectively, p = 0.7). T2 values averaged 31.2±10.3 and 36.6±12.2 ms; in thrombus from patients with and without adequate ST resolution (p = 0.09). After adjusting for diabetes and other baseline characteristics, lower T2 values were significantly associated with inadequate ST resolution (odds ratio for 1 ms increase 1.08, CI 95% 1.01–1.16, p = 0.027). Histology classified thrombus in 3 groups: coagulated blood (n = 38), fibrin rich (n = 9) and lipid-rich (n = 3). Thrombi composed mostly of coagulated blood were characterized as being of short (n = 10), intermediate (n = 15) or long evolution (n = 13), T2 values being 34.0±13.2, 31.9±8.3 and 31.5±7.9 ms respectively (p = NS). In this subgroup, T2 was significantly higher in specimens from patients with inadequate perfusion (35.9±10.3 versus 28.6±6.7 ms, p = 0.02). This can be of clinical interest as it provides information on the probability of adequate ST resolution, a surrogate for effective myocardial reperfusion.

## Introduction

ST-segment elevation acute myocardial infarction (STEMI), usually caused by thrombotic occlusion of a diseased coronary artery after erosion or rupture of an atherosclerotic plaque, is a leading cause of death in the western world [1]–[5]. Intracoronary thrombus is mostly composed of platelets and fibrin plus the remains of the atherosclerotic plaque, including fibrous cap, lipid core and/or hemorrhage, in the cases of plaque rupture [6]. It has been shown that a delay of days or even weeks may exist between plaque rupture and the onset of symptoms in STEMI patients [7]–[9].

Magnetic resonance imaging (MRI) is a noninvasive technique not involving ionizing radiation that has proven very useful in the evaluation of patients with ischemic heart disease. In addition to giving an accurate estimation of ventricular geometry and myocardial perfusion status, MRI can provide high resolution images of the coronary arteries which allow to identify the presence of intraluminal stenoses [10]. Moreover, MRI has been used to assess the composition and temporal evolution of thrombus in animal models of arterial damage [11], [12]. So far, clinical studies have focused on the analysis of the characteristics of the atherosclerotic plaque associated with the risk of rupture [13], [14]. Little is known, however, on the ability of MRI to characterize thrombus age and composition in the setting of STEMI.

The aim of the present work was to investigate if MRI analysis of the material obtained by thrombus aspiration during emergent percutaneous coronary intervention (PCI) in STEMI patients allowed to assess thrombus age and composition and also to correlate MRI findings with the success of primary PCI in terms of ST-segment resolution.

## Materials and Methods

The protocol was approved by the Hospital Vall d'Hebron Ethics Committee and all patients gave written consent to enter the study.

One hundred consecutive STEMI patients undergoing emergency PCI at our hospital were included. The inclusion criteria were all the following: a) symptoms suggesting acute myocardial ischemia lasting for more than 30 minutes and ST-segment elevation of more than 0.1 mV in two or more contiguous leads on the ECG; b) clinical indication of primary or rescue PCI; and c) use of a thrombus aspiration device during PCI. Of these patients, enough material for MRI analysis was obtained by thrombectomy in 59.

A 12-lead ECG was routinely recorded 120 min after primary PCI. Myocardial reperfusion was assessed according to the magnitude of ST-segment in this ECG trace in the lead with maximal ST elevation before PCI. Patients with ≥70% ST resolution were considered to have adequate myocardial reperfusion whereas no or less than 70% ST resolution was considered indicative of inadequate myocardial reperfusion [15]. Other relevant data were obtained from the clinical records.

After crossing the culprit lesion with the steerable guidewire, a 6-French Pronto V3 aspiration catheter (Vascular Solutions Inc., Minneapolis, MN) was advanced up to the distal vessel during continuous aspiration. Balloon dilation was performed before stenting only when necessary for stent delivery, but direct stenting after thrombectomy was the preferred technique. In all patients, intra coronary nitrates were given after restoration of anterograde flow to ensure maximal epicardial vasodilation. The use of bare metal or drug-eluting stents was left to the operator's discretion.

Pharmacologic treatment before PCI included aspirin (a loading oral dose of >250 mg), clopidogrel (a loading oral dose of either 300 or 600 mg), and intravenous unfractionated heparin (>5000 IU). Use of the glycoprotein IIb/IIIa inhibitor abciximab was left to the operator's discretion. Additional heparin was administered during the procedure according to the activated clotting time (target activated clotting time 250–300 s, or 200–250 s if glycoprotein IIb/IIIa inhibitors where administered).

Aspirated material was embedded in low-temperature gelling agarose (Sigma). Once the agarose solidified, it was transferred to a 9.4 T vertical bore magnet interfaced to a Bruker Avance 400 spectrometer (Bruker, Madrid, Spain) equipped with a 35-mm imaging probe. Sixty four images from a single slice were acquired with a spin-echo pulse sequence (m_msmevtr, Bruker) that allowed for the variation of both RT (Repetition Time) and ET (Echo Time); RT varied from 4000 to 138 ms in 8 steps while ET started at 6 ms and increased 6 ms each of the 8 steps up to 48 ms. Image acquisition lasted for approximately 30 min. Slice thickness was set to 500 µm while in plane resolution was 98×98 µm.

T1 and T2 (Spin-lattice and spin-spin relaxation) [16] time constants were calculated as follows: T1 was calculated by fitting the intensity of the area of interest (i.e. thrombus) in the 8 images obtained at different RT with the smallest ET to an exponential function “t1invacq” provided by Bruker; T2 was calculated by fitting the area of interest in the 8 images obtained with RT of 4000 ms to the equation Y = C exp(−t/T2) (Bruker). See figure S1 for a description of T2 calculation work flow.

Once the images were acquired, thrombi were fixed in formaldehyde, embedded in paraffin, cut in 5 µm sections and stained using standard procedures with hematoxilyn and eosin and Masson's techniques. Thrombi were classified as lipid-rich, fibrin-rich (at least 30% of the area occupied by fibrin) and as blood. In turn, blood thrombi were divided between short-, intermediate- and long- evolution time; this classification is comparable to that of fresh, lythic and organised thrombus described previously [17].

Statistical analysis was performed with SPSS 13.0 software (SPSS Inc., Chicago, IL). Continuous variables are reported as mean ± SD, and categorical variables as absolute numbers and percentages (given in brackets %). Comparisons between two continuous variables were performed by Student t tests or by the Mann-Whitney U test when appropriate and comparisons between two categorical variables were performed with chi-square tests or with the Fisher exact test. A multivariable logistic regression analysis was performed to assess the association between T1 and T2 values with myocardial reperfusion status after PCI after controlling by baseline predictors. The variables of interest were selected in a single step (enter method). Adjustement was made for age, active smoking and all baseline variables attained (p<0.2) with ST resolution at univariate analysis (sex, hypertension and diabetes mellitus). p values<0.05 were considered significant.

Receiver operating characteristics (ROC) analysis was performed according to the method of Hanley and McNeil [18] using MedCalc software (Mariakerke, Belgium).

## Results

Of the 59 patients with MR images of aspirated material, significant ST resolution indicating adequate myocardial reperfusion was observed in 31 (52%), and in the remaining 28 (48%) myocardial reperfusion was inadequate. Clinical, electrocardiographic, and angiographic data, and the results of MRI in both groups are summarized in table 1. The most conspicuous difference between the two groups was the higher prevalence of diabetes mellitus in the patients without adequate myocardial reperfusion (44% v 13% p = 0.003). None of the other characteristics analyzed reached statistical significant levels but there was a tendency for non-responders to have a higher risk profile. The magnitude of ST-segment elevation at presentation and the rates of complete occlusion of the culprit artery were similar in both groups. Compared with patients with optimal myocardial reperfusion, those with inadequate ST resolution 120 min after the procedure tended to have a lower TIMI (Thrombolysis in Myocardial Infarction) flow grade and had a significantly poorer TIMI angiographic blush grade immediately after PCI. We found no differences between rescue and primary PCI with respect to these variables.

**Table 1:** Clinical, electrocardiographic, and angiographic data, and results of MRI in both groups.

	Adequate myocardial reperfusion (n = 31)	Inadequate myocardial reperfusion (n = 28)	P value
Age, years	60±12	63±13	0.387
Male sex (%)	27 (87.1%)	20 (71.4%)	0.135
Active smoking (%)	20 (64.5%)	14 (50.0%)	0.260
Hypertension (%)	11 (35.5%)	16 (57.1%)	0.095
Diabetes mellitus (%)	3 (9.7%)	12 (42.9%)	0.003
Dyslipidemia (%)	19 (61.3%)	19 (67.9%)	0.599
N° of risk factors	1.7±0.9	2.2±1.1	0.076
Indication, primary PCI (%)	23 (74.2%)	21 (75.0%)	0.943
Indication, rescue PCI (%)	8 (25.8%)	7 (25.0%)	0.943
Time from onset of symptoms, h	5.5±2.6	6.5±5.0	0.325
Maximal ST elevation pre-PCI, mm	3.8±2.3	3.7±2.1	0.898
Initial TIMI flow grade = 0 (%)	29 (93.5%)	25 (89.3%)	0.661
Final TIMI flow grade = 3 (%)	28 (90.3%)	21 (75.0%)	0.223
Corrected TIMI frame Count	44.65±22.66	48.65±39.65	0.340
Final angiographic blush grade = 3 (%)	15 (51.7%)	6 (21.4%)	0.018
Maximal ST elevation post-PCI, mm	0.5±0.7	2.3±1.4	<0.001
% of ST resolution	89.4±11.2	32.1±40.0	<0.001
T1, ms	1248.0±572.8	1307.2±407.4	0.682
T2, ms	31.2±10.3	36.6±12.2	0.090

**Figure 1:**
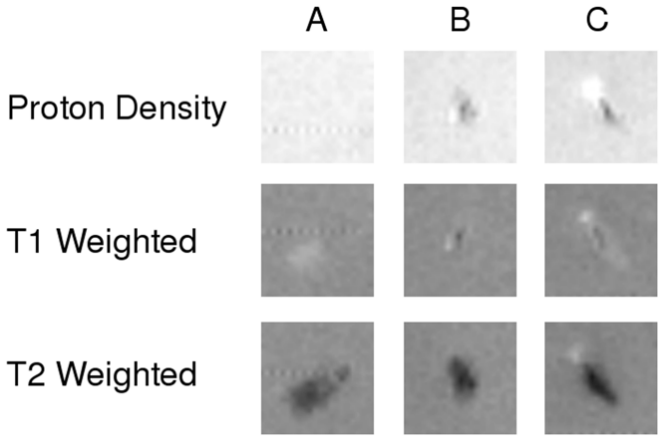
Typical MR Images of thrombus obtained at 9.4 T. Proton density (showing the amount of water), T1 and T2 weighted images (highlighting differences in T1 and T2 relaxation times respectively) of 3 samples obtained after thrombectomy. A) Thrombus, clotted blood, B) sample composed mainly of lipids and C) sample of mixed composition.

Examples of MR images of the aspirated material can be seen in figure 1. Thrombi showed differences in proton density, T1 and T2 weighted images. When T1 and T2 relaxation times were measured, we found a tendency for T2 values being longer in the group with poorer myocardial reperfusion (36.6±12.2 ms vs. 31.2±10.3 ms in patients with adequate ST resolution, p = 0.09) while T1 values were similar in the two groups.

Multivariable logistic regression analysis included age, sex, active smoking, hypertension, diabetes mellitus and MR variables. Only diabetes mellitus (odds ratio 8.14, 95% confidence limits 1.47–45.17, p = 0.016) and T2 value of the aspirated material (odds ratio for each ms of increase 1.08, 95% confidence limits 1.01–1.16, p = 0.027) were retained as independent predictors of inadequate myocardial reperfusion. Also, there was a trend for higher T2 values in patients not attaining optimal myocardial reperfusion at angiography (myocardial blush grade <3) than in the remaining patients (35.9±11.9 vs. 30.5±10.3 ms respectively, p = 0.119).

**Figure 2:**
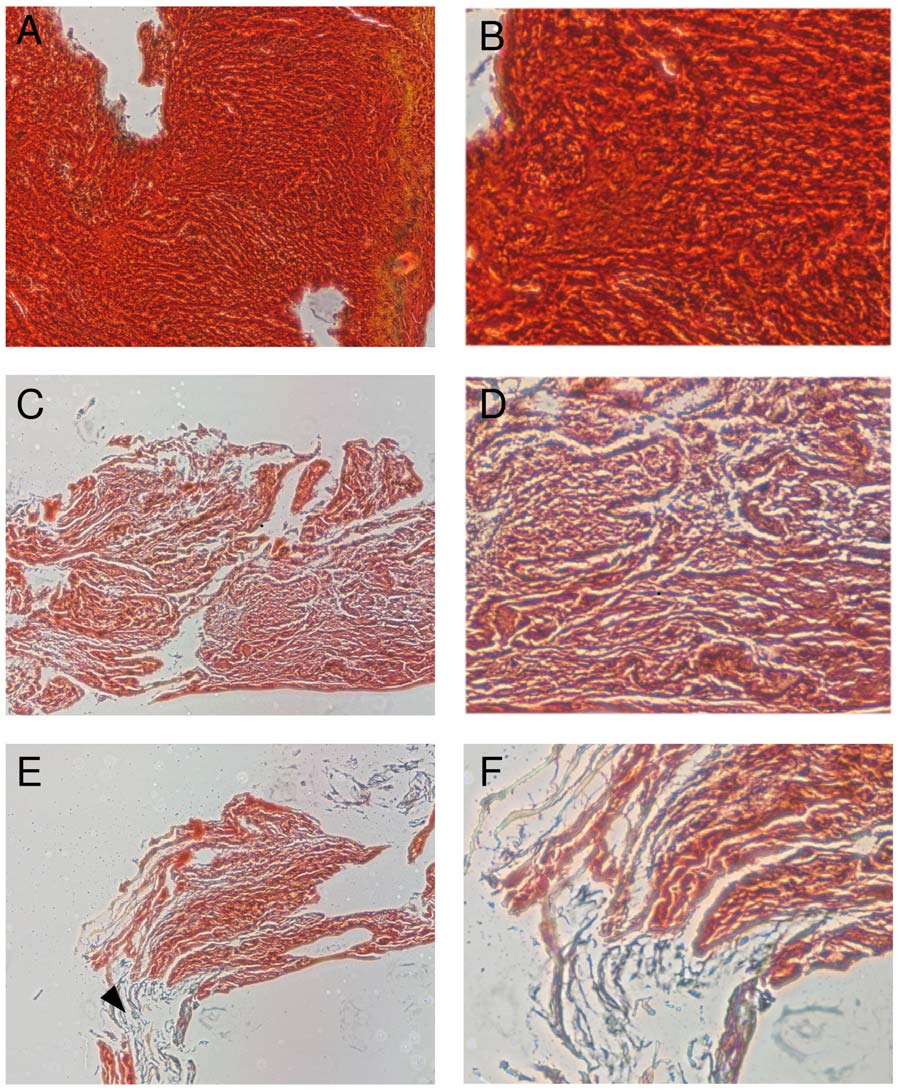
Histological analysis of thrombus. Histological images of blood thrombus of short (A, B), long (C, D) evolution time, and fibrin, marked with an arrowhead, rich (E, F) samples.

**Figure 3:**
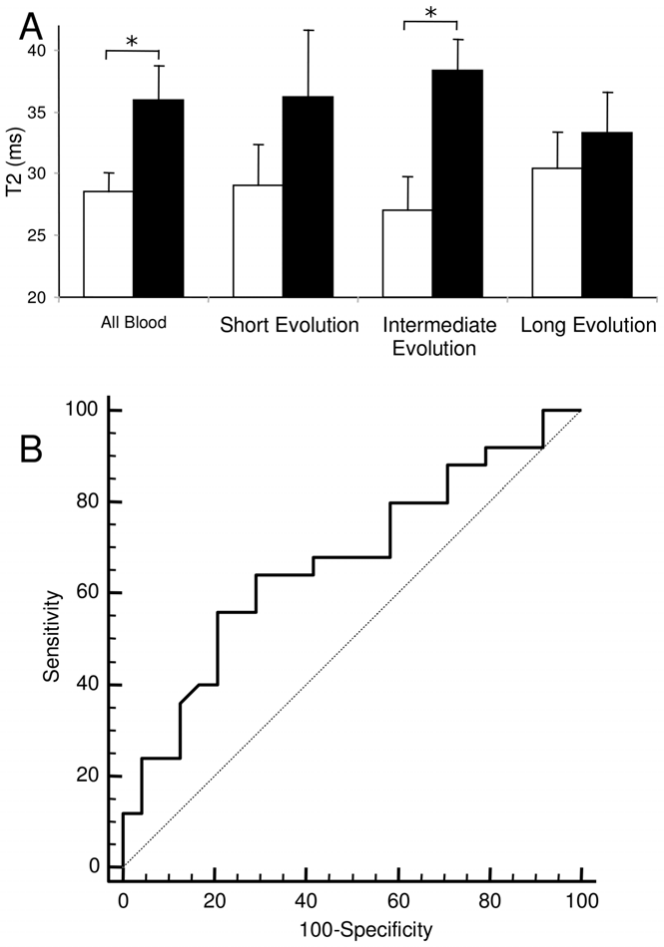
T2 values of coagulated blood. A, graph showing T2 values for coagulated blood. The first columns correspond to all blood samples, the others to groups divided according to their evolution time. White bars ST Resolution, full bars Non ST resolution. *statistical difference p<0.05. B corresponds to the receiver operating characteristic (ROC) curve obtained as described in the methods section. Area under the curve is 0.70, p = 0.03.

Histological analysis was completed in 50 specimens and showed three different patterns of thrombus composition, the most frequent consisting mostly of coagulated blood (n = 38), another characterized by a significant (at least 30% of the total area) content of fibrin (n = 9), and the most rarely seen being composed mostly of lipids (n = 3) (Figure 2). T2 values for each of these groups were, respectively, 32.4±1.6, 36.0±3.6 and 30.0±11.0 ms (p = NS) (Table 2). Thrombi composed mostly of coagulated blood were characterized as being of short (n = 10), intermediate (n = 15) or long evolution (n = 13). T2 values for each of these subgroups were 34.0±13.1, 31.9±8.3 and 31.5±7.9 ms respectively (p = NS). Interestingly, when the analysis was restricted to thrombi composed mostly of coagulated blood, T2 values were significatively different in the cases with and without ST resolution (28.5±6.7 and 35.9±10.3 ms, respectively, p = 0.02) (Figure 3). ROC curve analysis of these samples can be seen in Figure 3B; at a cut off value of 27.56 ms a sensitivity of 56 and specificity of 79.2% is achieved with an area under the curve of 0.70 (p = 0.03).

**Table 2:** T2 Values of aspirated material according to their hystological characteristics and ST resolution.

			ST Resolution		No ST Resolution		
	n	T2	n	T2	n	T2	P value
All Samples	59	32.89±10.19	31	31.20±10.3	28	36.6±12.2	0.09
Fibrin Rich	9	36.0±10.75	5	40.87±12.42	4	30.01±11.11	NS
Lipid Rich	3	30.0±19.1	2	19.18±4.43	1	51.8	NS
Blood	38	32.4±1.6	18	28.57±1.54	20	35.95±2.38	0.02

Statistics between ST resolution and no ST resolution are shown.

T1 values follow a similar tendency as T2 values; T1 of 1141.2±474.6 vs 1257.3±403.6 ms, (p = 0.44) in cases with and without ST resolution respectively without being statistical significant.

## Discussion

In the present study we have shown that MR analysis of the aspirated material obtained by thrombectomy during emergent coronary angioplasty in patients with STEMI, in particular T2 relaxation values, correlated with myocardial perfusion status as measured with ST segment resolution in the ECG.

The group of patients included in the study is representative of the consecutive cohort treated at our institution. We were able to obtain MR images from aspirated material of 59 out of 100 patients; this percentage is slightly lower than those reported in previous histological studies [9 and references cited therein]. These differences might be related to the fact that for MR imaging, a minimum sample volume of approximately 1 mm^3^ is needed; this is larger than the sample volume needed for histological studies.

In the study of all samples, logistic regression analysis showed that the presence of diabetes, as other studies found [19], was the most important factor in predicting inadequate myocardial reperfusion after angioplasty. The second most important predictor was T2 value, showing that T2 weighted images could be relevant in predicting the outcome of angioplasty. According to specificity and sensibility results obtained from ROC analysis, the measure of T2 on its own would not be enough as a clinical decision tool but would be able to provide relevant information in the everyday clinical practice.

When only blood thrombi were analyzed, T2 values were not able to differentiate between short, intermediate and long-term evolution but were significantly different between patients with and without ST resolution at univariate analysis. Data obtained in lipid- and fibrin-rich thrombus is inconclusive due to the low number of samples in these groups, thus it can be suggested that the differences seen in the whole population came mainly from coagulated blood samples.

Previous work on animal models has shown that both T1 and T2 values of thrombus change with time and that this change correlates with thrombus composition [20], [21]. MR imaging from large coronary plaques without thrombosis from a rabbit model obtained at 9.4 Tesla showed that T2 values were different according to plaque composition and fell within the range of values found in the present work [22]. It is possible that the addition of plaque material to the thrombus composition explain the observed differences in T2 values from aspirated material. Similar results were obtained on human carotid endarterectomy specimens obtained in the operating room [23].

We have found shorter T2 values in long-term evolved thrombi (described as organized in some reports). This correlates with better PCI angiographic results and myocardial perfusion in the present work. Similarly, Kramer and cols [9] have seen that organized thrombi show a lower degree of distal embolization, a predictor of poor outcome, than fresh thrombi.

MR angiography is able to detect coronary artery stenosis [24], [25]. Taking into account that by using different T1 and T2 weightings thrombus can be easily differentiated (Figure 1), it is possible that employing a series of pulse sequences would not only allow to detect coronary stenosis but also to gain insight into thrombus and plaque composition. At present, clinical angiography studies are done using a navigator-gated, cardiac-triggered, fat suppressed T1-W 3D gradient echo sequences with an in plane resolution of about 0.7 mm×1.00 mm [26] which is sufficient for the majority of cases studied.

T1 values get longer and show a narrower distribution range as magnetic field increases. This could be the reason why it has been reported that thrombus composition affects T1 at 1.5 Tesla [27] but we could not reproduce the findings at 9.4 Tesla. Shinnar and cols. [23] also reported that T1-weighted images added little additional information working at 9.4 Tesla and Sirol and cols. [21] reported that in the case of induced thrombosis in rabbit coronary arteries studied at 1.5 Tesla, signal intensity variation over time was significantly higher in T2 than in T1 weighted images. The same group working in the rabbit model described that T1 weighted black-blood sequencing allowed to differentiate the matrix from various stages of hemoglobin degrading products, indicating that T1-weighted images may further characterize the age of the thrombus [28]. Current literature suggests a T1-weighted 3D gradient echo for the visualization of coronary arteries; it can not be discarded that at standard clinical field strengths of 1.5–3 Tesla T1-weighted images could also be applied to differentiate between thrombus composition and thus to help predict angioplasty outcome in patients with acute coronary syndromes.

A limitation of this study is that all measures were conducted on material aspirated during angioplasty and that it is not clear up to what extent this may be reliably representative of the actual plaque/thrombus composition within the coronary artery. Also, a selection bias towards blood-rich thrombi vs. plaque-rich thrombi due to the amount of material needed for the analysis can not be discarded.

Although MRI assessment of thrombus age remains challenging, patients with adequate reperfusion tend to have lower T2 values (cut off value of 27.56 ms) as compared to those with inadequate reperfusion. Further studies are needed to investigate the clinical usage of MRI in patients with myocardial infarction for further risk assessment and prediction of interventional outcome.

## Supporting Information

Figure S1 **Work flow to measure relaxation time, first an image of the aspirated material is obtained at 8 different repetition and echo time conditions (A); the second step is to select regions of interest (ROI) within each thrombus (B).** To measure T2 the intensity of each ROI is plotted against echo time (C) and fitted to an exponential equation, for T1 measures (not shown) intensities are plotted against repetition time.
(TIF)
