# Supplementary figures and images for: MRI Discriminates Thrombus Composition and ST Resolution after Percutaneous Coronary Intervention in Patients with ST-Elevation Myocardial Infarction
Source: PLoS One. 2011 Apr 8;6(4):e18459. doi: 10.1371/journal.pone.0018459 (PMC3072998; doi:10.1371/journal.pone.0018459)

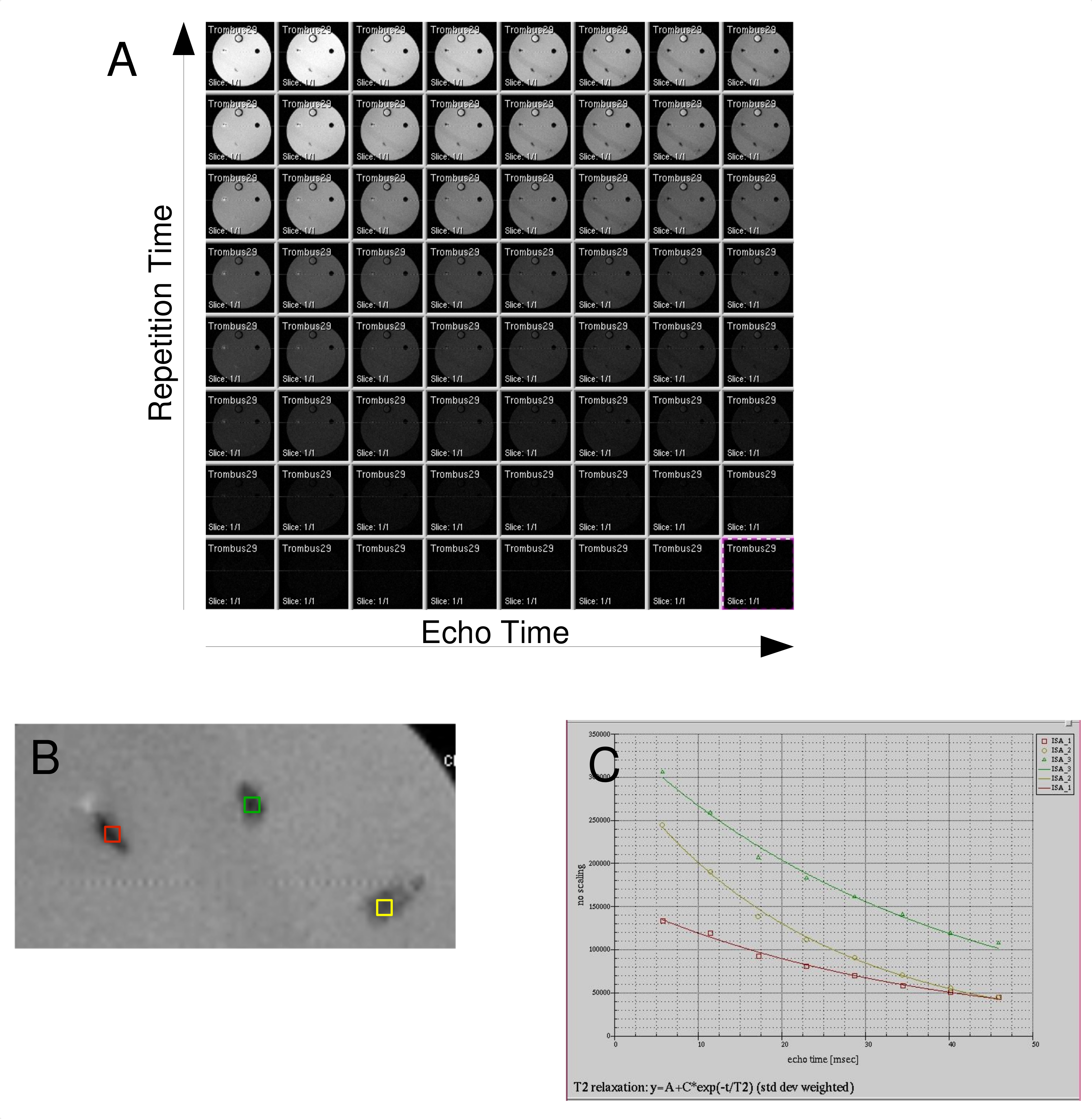

Supplement: Figure S1 — Work flow to measure relaxation time, first an image of the aspirated material is obtained at 8 different repetition and echo time conditions (A); the second step is to select regions of interest (ROI) within each thrombus (B). To measure T2 the intensity of each ROI is plotted against echo time (C) and fitted to an exponential equation, for T1 measures (not shown) intensities are plotted against repetition time. (TIF) [file pone.0018459.s001.tif]
